# Effect of Quaternary Ammonium Salt on Shear Bond Strength of Orthodontic Brackets to Enamel

**Published:** 2017-05

**Authors:** Hannaneh Ghadirian, Allahyar Geramy, Farhood Najafi, Soolmaz Heidari, Hamid Golshahi

**Affiliations:** 1 Assistant Professor, Dental Research Center, Dentistry Research Institute, Tehran University of Medical Sciences, Tehran, Iran; Department of Orthodontics, School of Dentistry ,Tehran University of Medical Sciences, Tehran, Iran; 2 Professor, Dental Research Center, Dentistry Research Institute, Tehran University of Medical Sciences, Tehran, Iran; Department of Orthodontics, School of Dentistry ,Tehran University of Medical Sciences, Tehran, Iran; 3 Assistant Professor, Department of Resin and Additives, Institute for Color Science and Technology, Tehran, Iran; 4 PhD Candidate, Department of Dental Biomaterials, School of Dentistry, Tehran University of Medical Sciences, Tehran, Iran; 5 Postgraduate Student, Department of Orthodontics, Tehran University of Medical Sciences, Tehran, Iran

**Keywords:** Dental Enamel, Shear Strength, Transbond XT Light Cure Adhesive Primer

## Abstract

**Objectives:**

This study sought to assess the effect of quaternary ammonium salt (QAS) on shear bond strength of orthodontic brackets to enamel.

**Materials and Methods:**

In this in vitro experimental study, 0, 10, 20 and 30% concentrations of QAS were added to Transbond XT primer. Brackets were bonded to 60 premolar teeth using the afore-mentioned adhesive mixtures, and the shear bond strength of the four groups (n=15) was measured using a universal testing machine. After debonding, the adhesive remnant index (ARI) score was determined under a stereomicroscope. Data were analyzed using one-way ANOVA.

**Results:**

The mean and standard deviation of shear bond strength of the control and 10%, 20% and 30% groups were 23.54±6.31, 21.81±2.82, 20.83±8.35 and 22.91±5.66 MPa, respectively. No significant difference was noted in shear bond strength of the groups (P=0.83). Study groups were not different in terms of ARI scores (P=0.80).

**Conclusions:**

The results showed that addition of QAS to Transbond XT primer had no adverse effect on shear bond strength of orthodontic brackets.

## INTRODUCTION

Prevention of enamel demineralization around orthodontic brackets is a challenge in orthodontic treatment [1]. Orthodontic brackets and appliances complicate oral hygiene practice for patients and enhance the accumulation of dental plaque on tooth surfaces [2]. Bracket bonding is routinely performed during fixed orthodontic treatment.

Despite numerous advantages, bracket bonding has a risk of debonding and plaque accumulation around brackets [3]. Bracket debonding may occur during orthodontic treatment and increases the duration of treatment and cost and necessitates additional patient visits [4]. Composite resins used for adhesive bracket bonding have a polymer matrix and can provide a suitable environment for proliferation of aerobic and anaerobic microorganisms [5]. Therefore, researchers are working on new formulations of bonding agents and composite resins with antibacterial activity to minimize the occurrence of white spot lesions. Several antibacterial agents have been added to orthodontic adhesives and composite resins to confer antibacterial activity. The antibacterial activity of quaternary ammonium salts (QASs) has been previously confirmed [6,7]. QAS is present in the formulation of Clearfil Protect Bond. QASs bond to bacterial cell membrane and cause its lysis and death [8]. Considering the significance of adequate bracket bond strength to enamel surface and minimizing the risk of occurrence of white spot lesions, this study aimed to assess the effect of QAS on shear bond strength of orthodontic brackets to enamel.

## MATERIALS AND METHODS

In this in vitro, experimental study, sample size was calculated to be 12 in each of the four groups for assessment of shear bond strength according to a previous study by Li et al, [9] assuming alpha=0.05, beta=0.2, effect size of 0.57 and standard deviation of 2.5 using one-way ANOVA. The current investigation was approved by the ethics committee of Tehran University of Medical Sciences (IR.TUMS.REC 1395.2909). The QAS compound that was synthesized and used in this study was methacryloxyethyl cetyl dimethyl ammonium chloride, which was added to Transbond XT light-cure adhesive primer (3M Unitek, Monrovia, CA, USA) [10]. Table 1 lists the constituents of primer and composite resin used in this study. A control group of pure primer was also considered.

**Table 1.** Constituents of primer and composite resin used in this study

**Product**	**Constituents**	**C.A.S No.**	**By wt%**
Primer (3M ESPE, St. Paul, MN, USA)	BisphonalA diglycidyl ether dimethacrylate (Bis GMA)	1565-94-2	45–55 trade Secret
	Triethylene Glycol Dimethacrylate	109-16-0	45–55 trade Secret
	Triphenylantimony	603-36-1	<1 trade Secret
	4- (Dimethylamino)-benzeneethanol	50438-75-0	<0.5 trade Secret
	Dl-camphorquinone	10373-78-1	<0.3 trade Secret
	Hydroquinone	123-31-9	<0.03 trade Secret
	Silane Treated Quartz	100402-78-6	70–80
Composite (3M ESPE, Monrovia, CA, USA)	Bisphenol A Diglycidyl Ether Dimethacrylate	1565-94-2	10–20
	Bisphenol A Bis (2-Hydroxiethyl ether) Dimethacrylate	24448-20-2	5–10
	Silane Treated Silica	68611-44-9	<2

The four study groups were as follows:

1. Control group containing pure primer
2. The 10% group, including 90wt% primer and 10wt% QAS
3. The 20% group, including 80wt% primer and 20wt% QAS
4. The 30% group, including 70wt% primer and 30wt% QAS

Different weight percentages were prepared using a digital scale (Santorius, Göttingen, Germany). For complete mixing, primer was incubated with QAS at 60°C for five minutes in an oven.

The containers were covered with foil to prevent light penetration. Next, the shear bond strength test was performed and the adhesive remnant index (ARI) score was determined.

### Shear bond strength test:

Sixty sound first premolars extracted for orthodontic purposes with no caries, cracks or restorations were cleaned from soft tissue residues and immersed in 0.5% chloramine T solution at 4°C for seven days. The teeth were randomly divided into four groups of 15, rinsed and dried. Next, 32% phosphoric acid (Scotchbond Universal etchant; 3M ESPE, St. Paul, MN, USA) was used for etching of the enamel according to the manufacturer’s instructions. The teeth were etched for 15 seconds, rinsed and dried to obtain a chalky white appearance. The four types of primer were used for bracket bonding (control and 10%, 20% and 30% concentrations of QAS). Transbond XT composite (3M Unitek, Monrovia, CA, USA) was applied on standard stainless steel edgewise premolar bracket base (American Orthodontics, Sheboygan, USA) with 0.018-inch slot size according to the manufacturer’s instructions with 12.62 mm^2^ bracket base area. Brackets were then bonded to the middle part of the buccal surface by applying equal force. After aligning the longitudinal axis of bracket parallel to the longitudinal axis of tooth, excess adhesive was removed by a scaler and light curing was performed using a light curing unit with 1200 mW/cm^2^ intensity. Each tooth was cured for 40 seconds from the mesial, distal, occlusal and gingival surfaces. All procedures were performed by the same operator. The samples were immersed in distilled water at 37°C for 24 hours.

**Fig. 1:**
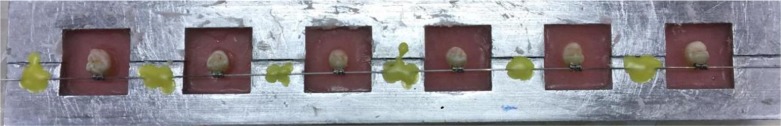
Teeth with acrylic blocks in molds

**Fig. 2:**
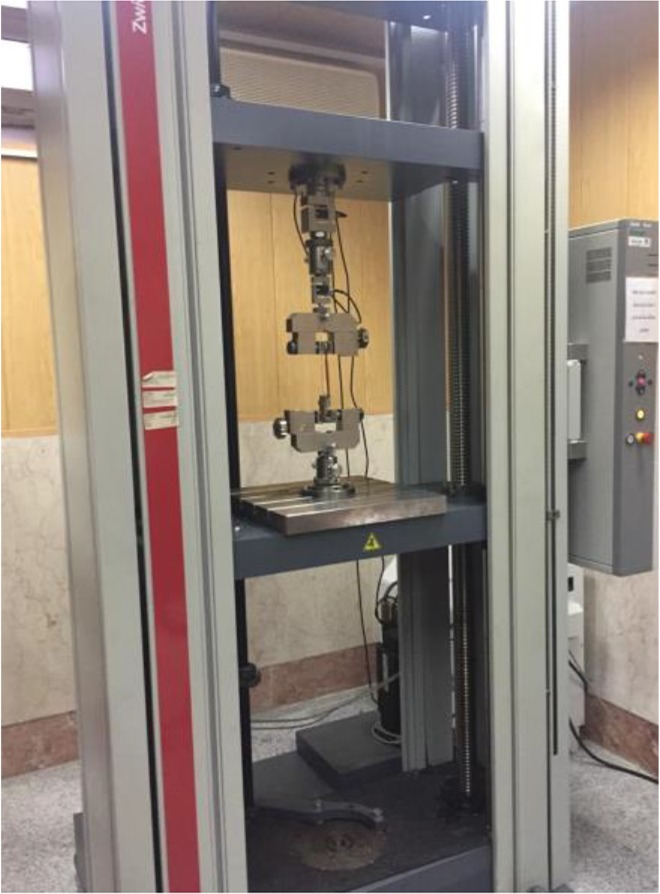
Universal testing machine

The samples were then subjected to 1500 thermal cycles between 5–55°C with a dwell time of 20 seconds and transfer time of 10 seconds [11]. The teeth were then mounted in molds measuring 2.5×2.5 cm. The internal surface of the mold was coated with petroleum jelly and the teeth were fixed using 16×22 inch rectangular stainless steel ligature wire. Each tooth was positioned at the center of the mold and the rectangular wire was fixed to the mold using sticky wax so that the teeth remained fixed when applying acrylic resin. Auto-polymerizing acrylic resin was applied to the mold and the teeth were embedded in acrylic to the level of their cementoenamel junction. After polymerization of acrylic resin, the teeth in acrylic blocks were separated from the mold (Fig. 1). The shear bond strength test was performed in Tehran University Dental Research Center. Universal testing machine (Zwick Roell, Ulm, Germany) was used for shear bond strength testing. The teeth were placed in the machine such that the bracket base was parallel to the load application vector. Load was applied in occlusogingival direction at a crosshead speed of 0.5 mm/minute to the bracket-tooth interface (Fig. 2). Load at debonding was recorded in Newtons (N) and converted to Megapascals (MPa) by dividing the load in Newtons by the bracket base surface area in square-millimeters (mm^2^).

### Determination of ARI score:

**Fig. 3:**
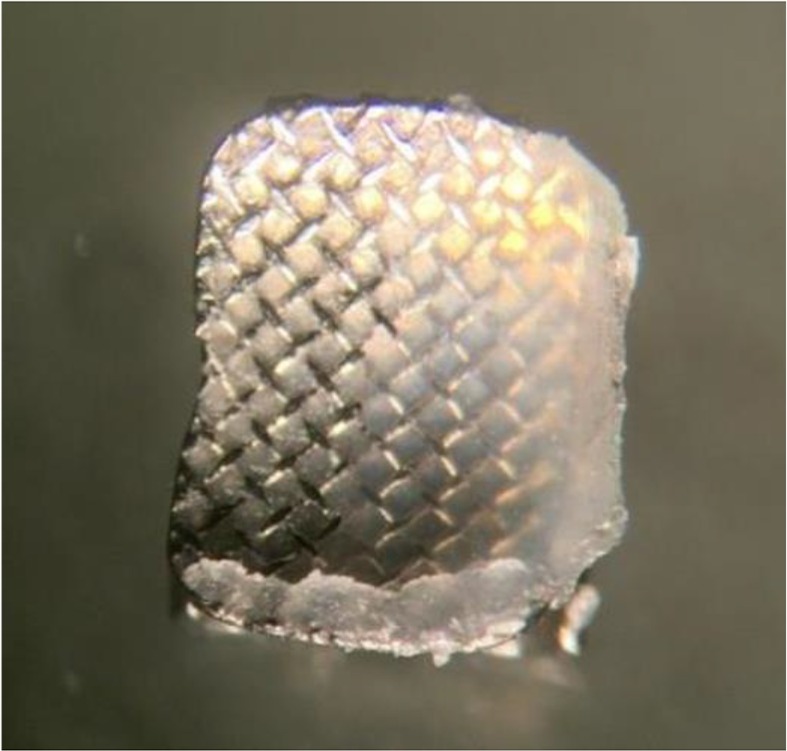
Debonded bracket under stereomicroscope at ×10 magnification

After debonding, each tooth and debonded bracket were evaluated under a stereomicroscope (SMZ800, Nikon, Tokyo, Japan) at ×10 magnification (Fig. 3). The ARI score was determined as follows:

Score 1. All adhesive remaining on the enamel surface
Score 2. 90% of adhesive remaining on the enamel surface
Score 3. 10–90% of adhesive remaining on the enamel surface
Score 4. Less than 10% of adhesive remaining on the enamel surface
Score 5. No adhesive remaining on the enamel surface [12]

## RESULTS

### Results of shear bond strength:

The mean bond strength values of the four groups are presented in Table 2. As shown, the highest bond strength was noted in the control group and the lowest in 20% group. According to ANOVA, the difference in shear bond strength was not significant among the four groups (P=0.83).

**Table 2.** Shear bond strength (MPa) of tested specimens in the four groups (n=15)

**Group**	**Minimum**	**Maximum**	**Mean**	**Standard deviation**
Control	11.89	37.63	23.54	6.31
10%	13.32	26.32	21.81	2.82
20%	4.87	35.96	20.83	8.35
30%	12.24	33.42	22.91	5.66

### Results of ARI score:

Table 3 shows the results of ARI scores. The Kruskal Wallis test showed no significant difference among the groups in terms of ARI scores (P=0.80).

**Table 3.** Adhesive remnant index (ARI) scores for the study groups

**ARI score**	**1**	**2**	**3**	**4**	**5**
Control	2	3	5	4	1
10%	3	3	3	4	2
20%	3	5	3	3	1
30%	2	3	4	4	2
Total	10	14	15	15	6

ARI: Adhesive remnant index

## DISCUSSION

This study sought to assess the effect of addition of QAS to Transbond XT primer on shear bond strength of orthodontic brackets to the enamel. No significant difference was noted in shear bond strength of the groups. Study groups were not different in terms of ARI scores. Orthodontic brackets enhance plaque accumulation in the oral cavity [13] and white spot lesions develop in 50 to 75% of patients under fixed orthodontic treatment [14]. Addition of antibacterial agents to composite resins and adhesives has been suggested as an effective approach for prevention of enamel demineralization during orthodontic treatment [15]. QASs have been suggested for this purpose due to their confirmed antibacterial activity. However, the effect of addition of this compound on physical and mechanical properties of adhesives and composite resin should also be evaluated.

A previous study showed that addition of QAS to composite resin caused no change in shear bond strength of brackets after one month [8]. This finding was in agreement with our results since we found no significant difference in shear bond strength of the groups containing QAS and the control group. No standard method exists for assessment of bracket bond strength to tooth structure because in the clinical setting, loads applied to brackets are complex and include a mixture of shear and tensile loads, which cannot be well simulated in vitro. However, shear bond strength test is often performed for this purpose [15] and was adopted in the current study as well. The results showed that by an increase in concentration of QAS, no significant reduction occurred in shear bond strength. Several factors affect the bracket bond strength to tooth structure including the type of adhesive, type of bracket, clinician’s expertise and patient’s cooperation [16].

Extracted human premolars were used in this study, which are ideal for assessment of bond strength since they have smooth buccal and lingual surfaces. Our results showed that the shear bond strength in presence of all concentrations of QAS was higher than the acceptable threshold of 6–8 MPa [17]. Thus, these concentrations may be used in the clinical setting with no adverse effect on bond strength, given that their other properties are also optimal for clinical use. This result was in agreement with that of Cheng et al. [18] who showed that addition of QAS to primer had no adverse effect on bond strength.

Zhang et al. [19] evaluated the effect of addition of silver nanoparticles and QAS to primer and adhesive and showed that addition of these compounds had no significant effect on bond strength and yielded a bond strength in the range of 30–35 MPa. However, Sodagar et al. [20] evaluated the addition of nano-chitosan particles to composite and showed that the composite containing 1% nano-chitosan had the highest and the composite containing 10% nano-chitosan had the lowest bond strength. They showed a reduction in bond strength by an increase in concentration of nano-chitosan particles. This result was in contrast to our findings, which may be due to the use of two different materials in the two studies. Poosti et al. [21] indicated that addition of titanium oxide significantly decreased the bond strength of composite to tooth structure. Their results were different from ours probably due to different materials used. Li et al. [9] evaluated the addition of QAS and silver nanoparticles to orthodontic primer and concluded that they had no adverse effect on bond strength. In this study, the effect of QAS on immediate bond strength was evaluated. Future studies are recommended to compare immediate and long-term bond strength after addition of QAS to orthodontic primer. Our study showed no significant difference in ARI scores of the four groups, which was in line with the results of a previous study [22]. However, our study had an in vitro design, and could not well simulate the clinical setting. In the oral environment, a combination of tensile, shear and torsional loads are applied and significant thermal changes, humidity, acidity and microbial plaque further complicate the situation, which cannot be ideally simulated in vitro. Thus, generalization of in vitro results to the clinical setting must be done with caution [23].

## CONCLUSION

Within the limitations of this study, the results showed that QAS had no adverse effect on bond strength of orthodontic brackets to enamel.
